# Epidemiology of pediatric uveitis and associated systemic diseases

**DOI:** 10.1186/s12969-021-00516-2

**Published:** 2021-04-01

**Authors:** Yoonkyeom Shin, Ji-Man Kang, Junwon Lee, Christopher Seungkyu Lee, Sung Chul Lee, Jong Gyun Ahn

**Affiliations:** 1 Department of Pediatrics, Severance Children’s Hospital, Yonsei University College of Medicine, 50-1 Yonsei-ro, Seodaemun-gu, Seoul, 03722 Korea; 2 Institute for Immunology and Immunological Diseases, Yonsei University College of Medicine, Seoul, Korea; 3 Department of Ophthalmology, Institute of Vision Research, Severance Hospital, Yonsei University College of Medicine, Seoul, Korea

**Keywords:** Children, Juvenile idiopathic arthritis, Antinuclear antibodies, HLA-B27

## Abstract

**Background:**

The early detection of uveitis associated with systemic inflammatory disease in children is important for proper treatment and prognosis. However, the diagnosis may be delayed because of difficulties in childhood examinations and early minor systemic symptoms. The objective of our study was to identify the pattern of childhood uveitis and investigate the frequency and clinical features of rheumatic diseases in pediatric patients with uveitis.

**Methods:**

This retrospective observational study reviewed the medical records of children (age ≤ 18 years) with uveitis at a Korean tertiary hospital between January 2005 and December 2018. Data collected included the age at onset of uveitis, sex, anatomic location of ocular inflammation, comorbid disease (including systemic inflammatory disease), ocular complications, relevant laboratory data, and treatment. Fisher’s exact test was used to compare categorical variables and the Mann–Whitney U test was used to compare continuous variables. A *p*-value of < 0.05 was considered statistically significant.

**Results:**

A total of 155 pediatric patients with uveitis were included in this study. The median age at diagnosis was 13.0 years (interquartile range, 9.5–16.0 years). The male-to-female ratio was 1.09. The process was unilateral in 51.6% of children. Anterior uveitis, panuveitis, intermediate uveitis, and posterior uveitis represented 51.6, 26.5, 6.5, and 1.9% of the cases, respectively. Idiopathic uveitis (65.2%) was the most frequent type of uveitis. Systemic rheumatic disease associations were responsible for 28.4% of the cases, among which juvenile idiopathic arthritis (JIA) was the most frequent cause (14.8%). Human leukocyte antigen (HLA)-B27 and antinuclear antibody (ANA) positive rates were significantly higher in patients with JIA than in those with idiopathic uveitis (*p* = 0.006 and *p* = 0.007, respectively).

**Conclusions:**

Approximately one-third of children with uveitis in Korea have a systemic rheumatic disease, of which JIA accounts for the majority of cases. HLA-B27 and ANA can serve as risk factors for JIA-associated uveitis.

**Supplementary Information:**

The online version contains supplementary material available at 10.1186/s12969-021-00516-2.

## Background

Uveitis is a condition characterized by the inflammation of the uveal components of the eye, namely the iris, choroid, and retina. Uveitis in the pediatric population accounts for 2 to 14% of all patients with uveitis [1, 2]. In South Korea, the overall incidence and prevalence of uveitis were 10.6 per 10,000 person-years and 17.3 per 10,000 persons, respectively, during 2007–2013. However, in the Korean population, the incidence of uveitis was the lowest among children aged 0–9 years (1.0 per 10,000 person-years) and 10–19 years (4.2 per 10,000 person-years) [3]. Although the incidence of uveitis is known to be lower in children than in adults, the former experience more severe ocular comorbidities that affect their quality of life [4–6].

Uveitis usually occurs as an isolated illness without specific cause but may also occur in association with many other medical conditions, especially systemic inflammatory diseases. The systemic immune-mediated causes of uveitis include spondyloarthritis, inflammatory bowel disease, Behçet disease, and juvenile idiopathic arthritis (JIA) [7]. The systemic inflammatory causes of uveitis in children are somewhat different from those in adults. In children, JIA and Kawasaki disease, which are not common in adults, have a causal association with uveitis [5, 8]. The early detection of pediatric uveitis associated with systemic inflammatory disease is important for proper treatment and prognosis. However, the systemic symptoms in the early stages may be mild, making it difficult to recognize these diseases. Therefore, it is necessary to understand the epidemiology of the clinical characteristics and frequency of rheumatoid diseases that are frequently associated with pediatric uveitis.

Although several reports have described the epidemiological profile of pediatric uveitis worldwide, no reports have described the situation in Korea. In the present study, we analyzed the clinical features and systemic immune-mediated associations in children with uveitis. We also determined whether the clinical and laboratory findings differed between pediatric patients with uveitis with and without rheumatoid disease.

## Methods

### Study design and data collection

This retrospective observational study was conducted at a tertiary referral hospital with pediatric rheumatologists and ophthalmologists serving the pediatric rheumatology service from January 2005 to December 2018. We reviewed the medical charts of all children with uveitis aged ≤18 years. The research was approved by the institutional review board (no. 4–2020-0142) of the Yonsei University Health System. The requirement for informed consent was waived due to the retrospective nature of the study.

The collected data included the age at onset of uveitis, sex, anatomic location of ocular inflammation, comorbid disease (including systemic inflammatory disease), ocular complications, relevant laboratory data, and treatment. Uveitis was divided into four major categories based upon the etiology of inflammation: systemic immune-mediated diseases, syndromes confined primarily to the eye, masquerade syndrome, and infection. The term “idiopathic uveitis” was used when a patient with uveitis did not fit into any of the above well-defined diagnostic categories and lacked an identifiable infectious etiology or apparent associated systemic disease. The anatomic location, duration, and course of uveitis was classified according to the Standardization of Uveitis Nomenclature [9]. Laboratory tests were conducted to evaluate the antinuclear antibody (ANA), rheumatoid factor, human leukocyte antigen (HLA)-B51, HLA-B27 levels, and serologies for toxoplasma, herpes simplex virus, and/or herpes zoster virus. An ANA titer of ≥1:80 by fluorescent antibody testing was considered positive. Treatments specific for uveitis were recorded, including corticosteroids (local and systemic), synthetic disease-modifying antirheumatic drugs (DMARDs; methotrexate, cyclosporine, colchicine, and mycophenolate), and biological DMARDs (adalimumab, etanercept, and infliximab). The treatment included only for uveitis except for control of systemic disease. The subtypes of JIA were classified using the International League of Associations for Rheumatology (ILAR) criteria [10]. In comparison with other studies, spondyloarthritis was considered an enthesitis-related arthritis (ERA) among the JIA subtypes [11].

### Statistical analysis

Descriptive statistics for continuous and categorical variables are presented as medians with interquartile ranges (IQR) and as frequencies and percentages, respectively. The Pearson chi-square test (Fisher’s exact test) and Mann–Whitney U test were used to compare categorical and continuous variables, respectively. A *p*-value of < 0.05 was considered statistically significant. All analyses were performed using the SPSS software, version 25 (SPSS Inc., Chicago, IL, USA).

## Results

### Patient characteristics

During the 14-year study period, 205 patients were identified using diagnostic codes in their medical records. Fifty patients were excluded based on medical chart reviews, which ultimately determined that they did not have uveitis, although they had been referred to our medical center from other clinics under a suspicion of uveitis. Therefore, 155 patients were included in the present study: 81 boys (52.3%) and 74 girls (47.7%). The median age at diagnosis was 13.0 years (IQR, 9.5–16.0 years). The median ages of the male and female patients were 13.0 years (IQR, 10.0–16.0 years) and 12.0 years (IQR, 8.3–16.0 years), respectively.

### Etiologies of pediatric uveitis

The causes of pediatric uveitis are summarized in Table 1. Among the 155 children with uveitis, the most common cause of uveitis was idiopathic (*n* = 101, 65.2%), followed by systemic immune-mediated disease (*n* = 44, 28.4%), syndrome confined primarily to the eye (*n* = 5, 3.2%), masquerade syndrome (n = 4, 2.6%), and infection (*n* = 1, 0.7%). The five most common systemic immune-mediated etiologies were JIA (*n* = 23, 14.8%), Behçet disease (*n* = 10, 6.5%), Kawasaki disease (*n* = 3, 1.9%), Vogt–Koyanagi–Harada syndrome (n = 3, 1.9%), and tubulo-intestinal nephritis (n = 2, 1.3%). JIA accounted for 45.5% of all the systemic cases. Among the 23 patients with JIA, oligoarthritis was the most common subtype (*n* = 13, 56.5%), followed by ERA (*n* = 8, 34.8%) (Additional file 1).

**Table 1:** Etiologies of pediatric uveitis

Etiology	Total N (%)	Categories	N (%)	Median age at uveitis diagnosis, y (IQR)	Systemic treatment, N (%)	Median follow-up, m (IQR)
Idiopathic	101 (65.2)			13.0 (10.0–16.0)	39 (38.6)	16.0 (2.0–53.0)
Systemic immune-mediated disease	44 (28.4)	Juvenile idiopathic arthritis	23 (14.8)	11.0 (6.5–16.0)	20 (87.0)	72.0 (2.5–111.0)
		Behçet disease	10 (6.5)	15.0 (13.3–16.8)	9 (90.0)	120.0 (67.5–132.0)
		Kawasaki disease	3 (1.9)	11.0 (11.0–12.0)	3 (100)	15.0 (14.5–33.5)
		Vogt–Koyanagi–Harada syndrome	3 (1.9)	16.0 (15.0–17.0)	2 (66.7)	36.0 (29.0–60.0)
		Tubulo-intestinal nephritis	2 (1.3)	11.5 (11.3–11.8)	2 (100)	49.5 (38.3–60.8)
		Systemic lupus erythematosus	1 (0.7)	6	1 (100)	180
		Kikuchi disease	1 (0.7)	17	1 (100)	35
		Inflammatory bowel disease	1 (0.7)	13	1(100)	42
Syndrome confined primarily to the eye	5 (3.2)	Trauma	3 (1.9)	12.0 (8.0–16.0)	1 (33.3)	9.0 (7.5–40.5)
		Posner–Schlossman syndrome	1 (0.7)	18	1 (100)	0.3
		Sympathetic ophthalmia	1 (0.7)	9	1 (100)	3
Masquerade syndrome	4 (2.6)	Langerhans cell histiocytosis	2 (1.3)	13.0 (11.0–17.0)	1 (50.0)	95.5 (77.3–113.8)
		Acute lymphocytic leukemia	1 (0.7)	18	1 (100)	60
		Hydroa vacciniforme-like lymphoma	1 (0.7)	6	1 (100)	16
Infection	1 (0.7)	Varicella zoster virus	1 (0.7)	17	0 (0.0)	21

*m* months

### Clinical characteristics of children with uveitis

The patients’ clinical characteristics are shown in Table 2. Uveitis was unilateral in 80 patients (51.6%) and bilateral in 75 patients (48.4%). Anterior uveitis was the most common anatomical form (*n* = 80, 51.6%), followed by panuveitis (*n* = 41, 26.5%), intermediate uveitis (*n* = 10, 6.5%), and posterior uveitis (*n* = 3, 1.9%). All patients initially controlled their eye inflammation with topical steroid therapy (*n* = 155, 100%). Systemic therapy was used in 78 patients (50.3%). Systemic treatments included systemic corticosteroids in 78 patients (50.3%), synthetic DMARDs in 48 patients (31.0%), and biological DMARDs in 12 patients (7.7%). Ocular complications such as band keratopathy, posterior synechiae, cataract, ocular hypertension, and cystoid macular edema occurred in 60 patients (38.7%).

**Table 2:** Characteristics of children with uveitis

Characteristics	N (%)
Sex	
Male	81 (52.3)
Female	74 (47.7)
Median age at uveitis diagnosis, y (IQR)	13 (9.5–16.0)
Type	
Unilateral	80 (51.6)
Bilateral	75 (48.4)
Location	
Anterior	80 (51.6)
Intermediate	10 (6.5)
Posterior	3 (1.9)
Panuveitis	41 (26.5)
Unknown	21 (13.5)
Treatment	
Topical steroid	155 (100.0)
Systemic steroid	78 (50.3)
Synthetic DMARDs	48 (31.0)
Biological DMARDs	12 (7.7)
Ocular complications	60 (38.7)

*y* years, *IQR* interquartile range, *DMARDs* disease-modifying antirheumatic drugs

### Comparison of clinical features among groups according to the causes of uveitis

The demographic, clinical, and laboratory findings and ocular complications of patients with systemic immune-mediated and idiopathic uveitis are shown in Table 3. There were no significant differences in these findings between the two groups, with the exception of uveitis treatment. Children with systemic inflammatory disease-associated uveitis had a significantly higher frequency of systemic treatment than those with idiopathic uveitis (*p* < 0.001 for systemic steroids, synthetic DMARDs, and biological DMARDs).

**Table 3:** Comparison of characteristics of patients with uveitis

Characteristics	(1) Uveitis with Systemic immune-mediated disease, *n* = 44	(2) JIA-related uveitis, *n* = 23	(3) Idiopathic uveitis, *n* = 101	*p-value* [(1) vs. (3)]	*p-value* [(2) vs. (3)]
Sex, N(%)					
Male	25 (56.8)	15 (65.2)	48 (47.5)	0.303	0.126
Female	19 (43.2)	8 (34.8)	53 (52.5)		
Median age at uveitis diagnosis, y (IQR)	13.0 (10.0–16.0)	11.0 (6.5–16.0)	13.0 (10.0–16.0)	0.914	0.251
Laboratory findings					
HLA-B27 tested, N	27	16	43		
Positive, N (%)	8 (29.6)	7 (43.8)	5 (11.6)	0.059	0.006
HLA-B51 tested, N	18	5	41		
Positive, N (%)	5 (27.8)	1 (20.0)	7 (17.1)	0.347	0.871
RF tested, N	41	23	71		
Positive, N (%)	5 (12.2)	3 (13.0)	5 (7.0)	0.357	0.370
ANA tested, N	40	23	66		
Positive, N (%)	16 (40.0)	13 (56.5)	17 (25.8)	0.125	0.007
Type, N (%)					
Unilateral	18 (40.9)	15 (65.2)	54 (53.5)	0.164	0.306
Bilateral	26 (59.1)	8 (34.8)	47 (46.5)		
Location, N (%)					
Anterior	22 (50.0)	13 (56.5)	52 (51.5)	0.633	0.779
Intermediate	1 (2.3)	1 (4.4)	9 (8.9)		
Posterior	1 (2.3)	0 (0.0)	2 (2.0)		
Panuveitis	14 (31.8)	5 (21.7)	25 (24.8)		
Unknown	6 (13.6)	4 (17.4)	13 (12.9)		
Treatment, N (%)					
Topical steroid	44 (100.0)	23 (100.0)	101 (100.0)		
Systemic steroid	37 (84.1)	20 (87.0)	35 (34.6)	< 0.001	< 0.001
Synthetic DMARDs	31 (70.5)	17 (73.9)	15 (14.9)	< 0.001	< 0.001
Biological DMARDs	11 (25.0)	6 (26.1)	0 (0.0)	< 0.001	< 0.001
Ocular complications, N (%)	21 (47.7)	11 (47.8)	35 (34.7)	0.137	0.238

*y* years, *JIA* juvenile idiopathic arthritis, *IQR* interquartile range, *HLA* human leukocyte antigen, *RF* rheumatoid factor, *ANA* antinuclear antibody, *DMARDs* disease-modifying antirheumatic drugs

The demographic, clinical, and laboratory findings and ocular complications of patients with JIA, the most common systemic immune-mediated etiology of uveitis, were compared with those of patients with idiopathic uveitis (Table 3). The proportions of positive HLA-B27 and ANA cases were significantly higher among patients with JIA-related uveitis than among those with idiopathic uveitis (43.8% vs. 11.6%, *p* = 0.006 and 56.5% vs. 25.8%, *p* = 0.007, respectively). Patients with JIA-related uveitis were significantly more likely to receive systemic treatment than those with idiopathic uveitis (*p* < 0.001 for systemic steroid, synthetic DMARDs, and biological DMARDs). There were no significant differences between the two groups in other demographic, clinical, and laboratory findings and ocular complications.

## Discussion

To our knowledge, this was the first large-scale study to investigate the epidemiology of pediatric uveitis and the associated systemic inflammatory diseases in Korea. In our study, systemic rheumatic disease was confirmed in 28.4% of children with uveitis, and this was the second leading cause of pediatric uveitis after idiopathic uveitis. Of the identified systemic rheumatic diseases, JIA (14.8%) was the most common, followed by Behçet disease (6.5%), Kawasaki disease (1.9%), and Vogt–Koyanagi–Harada syndrome (1.9%). In most previous large studies of pediatric uveitis published after 2000, JIA was reported as the most common systemic disease (Table 4) [1, 5, 6, 12–14, 16].

**Table 4:** Comparison of large studies of pediatric uveitis in other countries

Study	N	Location	Etiology (%)						Anatomic distribution of uveitis (%)			
			Idiopathic^f^	JIA	Behçet disease	Kawasaki disease	VKH	Infection	Anterior	Intermediate	Posterior	Panuveitis
Edelsten et al. (2003) [5]	249	UK	44	47	(−)	(−)	(−)	2	70	0	30	0
Kadayifcilar et al. (2003) [12]	219	Turkey	36^a^	15.0	11.0	0	0.5	30.1	43.4	11.9	31	13.7
Boer et al. (2003) [13]	123	Netherlands	53.7	20	0	0	0	13.3	36	24	19	21
Rosenberg et al. (2004) [6]	148	USA	41.3^b^	23	0.7	0	0.7	24.3	30.4	27.7	23.7	18.2
Benezra et al. (2005) [1]	276	Israel	25.4	15.3	4.7	0	1.1	33.3	13.4	41.7	14.1	30.8
Kump et al. (2005) [14]	269	USA	51.7^c^	33.1	0.4	7.4	0.7	5.2	56.9	20.8	6.3	16
Khairallah et al. (2006) [15]	64	Tunisia	49.9	6.2	6.2	0	1.5	25	31.3	31.3	20.3	17.2
Smith et al. (2009) [16]	527	USA	45.9^d^	21.6	1.9	0	2.8	11.4	44.6	28	14.4	12.9
Ozdal et al. (2012) [17]	121	Turkey	40.4^e^	10.7	16.5	0	0.8	21.5	31.4	25.6	24.8	18.2
Abd El Latif et al. (2019) [18]	413	Egypt	28.6	6.7	6.8	0	1.9	36.3	27.1	30	18.6	24.2
Keino et al. (2017) [19]	64	Japan	57.8	0	3.1	1.6	1.6	6.3	56.3	0	15.6	28.1
Current study	155	Korea	65.2	14.8	6.5	1.9	1.9	0.7	51.6	6.5	1.9	26.5

*JIA* juvenile idiopathic arthritis, *VKH* Vogt–Koyanagi–Harada syndrome, *UK* United Kingdom, *USA* United States of America

^a^Idiopathic uveitis includes pars planitis (11.8%) and idiopathic intermediate uveitis (24.2%)

^b^Idiopathic uveitis includes pars planitis (14.9%) and idiopathic uveitis (26.4%)

^c^Idiopathic uveitis includes pars planitis (did not specify the number of patients of pars planitis)

^d^Idiopathic uveitis includes pars planitis (17.1%) and idiopathic uveitis (28.8%)

^e^Idiopathic uveitis includes pars planitis (23.9%) and idiopathic uveitis (16.5%)

^f^Other studies did not distinguish between pars planitis and idiopathic intermediate uveitis

A report from the United Kingdom identified JIA-related uveitis (47%) as the most common cause of pediatric uveitis [5]. JIA-associated uveitis accounted for 21.6–33.1% of all cases of pediatric uveitis in three studies conducted in the United States [6, 14, 16]. In contrast, a study in Japan observed no JIA-related pediatric uveitis, and this discrepancy was attributed to the low prevalence of JIA itself in Japan relative to North America and Europe [19]. The overall prevalence of JIA is also known to be lower in African-American and Asian populations [20]. In studies conducted in Tunisia and Egypt, JIA was the etiology in 6.2 and 6.7% of the evaluated cases of pediatric uveitis, respectively, and both proportions were lower than those in the United States and United Kingdom. A population-based cohort study conducted in Taiwan showed that while the incidence of JIA was low, the incidence of JIA-associated uveitis had increased from 0.16 cases per 100,000 children in 1999 to 0.37 cases per 100,000 children in 2009 [21]. Although the prevalence of JIA has not been investigated in Korea, the identification of this disease as the most common rheumatic cause of pediatric uveitis in our study suggests that JIA should be included in the differential diagnosis of the etiology of childhood uveitis, even in countries with a low prevalence of JIA. In our study, oligoarticular JIA and ERA were the most common subtypes in patients with JIA-associated uveitis, accounting for 91.3% of all JIA cases. These results are consistent with those of earlier studies [22–24]. Pediatric uveitis has been reported to occur in 15–25% of children with oligoarthritis and in 3–7% of children with ERA [21, 23, 25–27]. Screening JIA-associated uveitis guidelines of the American Association of Pediatrics (AAP) [28, 29] and Single Hub and Access point for pediatric Rheumatology in Europe (SHARE) [30] continue to undergo revision but are based on four risk factors for developing uveitis: the category of arthritis, age at onset of arthritis, the presence of ANA positivity, and duration of the disease. Oligoarticular JIA in children is one of the risk factors of uveitis; therefore, more frequent ophthalmological screening is recommended for children with oligoarticular JIA than those with other JIA subtypes [28]. ERA was not yet a risk factor in those guidelines, but this type was the second most common type of JIA-related uveitis. Therefore, children with oligoarthritis and ERA should undergo ophthalmological screenings at the time of these diagnoses and should be followed up more carefully than those with other JIA subtypes.

In the present study, anterior uveitis (51.6%) was the most common form of involvement, followed by panuveitis (26.5%), intermediate uveitis (6.5%), and posterior uveitis (1.9%). Anterior uveitis was also the most common form of involvement in almost all series of European countries, North America, and Asia, with a prevalence range of 30.4–70% [5, 6, 13, 14, 16, 19]. In particular, JIA-associated anterior uveitis was identified as the leading etiology in series reported from Northern Europe and North America; this entity is less frequent in Africa and the Middle East, where intermediate uveitis is dominant [1, 5, 6, 14–16, 18].

Panuveitis (26.5%) accounted for a relatively high proportion of cases in our study compared to the rates in the United Kingdom and North America (0–18.2%). In addition, as shown in Table 4, relatively high proportions of panuveitis were reported in Turkey, Japan, and Israel (18.2, 28.1, and 30.8%, respectively) [1, 17, 19]. These countries also reported a relatively high prevalence of Behçet disease-associated uveitis, which usually presents as panuveitis. Although it is well known that the anatomical locations of uveitis vary by region and race, one reason for these differences may include variability in the prevalence of other systemic diseases that cause uveitis by region and race. These findings are also supported by the fact that toxoplasmosis-associated uveitis, the most common cause of posterior and infectious uveitis, was observed more frequently in Turkey [12, 17]. In contrast, our study results showed that only 1.9% of cases were posterior uveitis; this is probably due to the low incidence of infectious uveitis in Korean children. The Korea Disease Control and Prevention Agency has reported the number of toxoplasmosis annually [31]. About 5 to 20 cases of toxoplasmosis infection have been reported annually, regardless of age. Of those cases, there is none or only one case per year in pediatrics. Even in ocular toxoplasmosis among adults, there are limited data on the incidence, clinical characteristics, and disease course in Korea compared to that in other countries [32]. This is because Korea is a low-endemic area of *Toxoplasma gondii*.

In the present study, intermediate uveitis accounted for 6.5% of all cases of childhood uveitis, compared to 20.8–27.7% of cases at referral practices in North America [6, 14, 16]. The association of intermediate uveitis with systemic disease is very rare in children. Associations between idiopathic intermediate uveitis and HLA-DR2 and HLA-DR15 have been reported, which suggests an immunogenetic predisposition. These HLA types are less common in Japan and Korea than in predominantly Caucasian countries, which may explain why the incidence of intermediate uveitis is low in Korea. In addition to the above-mentioned differences in the prevalence of systemic diseases in uveitis for each race, we postulate that racially distinctive genetic factors may also influence the location of the affected uveal tract.

In the current study, JIA-associated and idiopathic uveitis cases differed significantly in terms of ANA and HLB-B27. These results are consistent with those of previous studies. In particular, ANA in oligoarthritis is known to be associated with pediatric uveitis [22–24, 28]. The specific role of ANA in pediatric uveitis remains unknown, although reactivity to histones has been reported to occur more frequently in patients with JIA-associated uveitis than in those with JIA alone [33]. The early detection and prompt treatment of patients with JIA-associated uveitis is associated with a good visual prognosis. However, the uveitis seen in JIA is characterized by typical chronic asymptomatic anterior uveitis. Therefore, the presence of ANA should be determined even in patients with JIA with no immediately evident ocular symptom, and ANA-positive children should be monitored more closely for the development of uveitis than ANA-negative children as the AAP guideline recommends [28, 29].

HLA-B27 is an important part of the inclusion criteria used in the definition of ERA based on the ILAR criteria [10]. HLA-B27 also has a strong association with ankylosing spondylitis (AS), which is notable considering that ERA is considered to be the pediatric counterpart of adult AS [11]. Although there is limited evidence for genetic susceptibility to uveitis in JIA, acute anterior uveitis is known to be associated with the HLA-B27 class I gene [34, 35]. In a Mexican study, polymorphisms in the HLA-linked LMP2 locus were associated with a higher risk of acute uveitis development in an HLA-B27-positive population with AS [36]. In children with ERA, uveitis uveitis is often noted in 7–15% of patients as acute, recurrent anterior inflammation with eye pain, photophobia, or conjunctival injection, as opposed to the typical asymptomatic anterior uveitis seen in JIA patients with oligoarthritis [25, 28]. Therefore, children with acute and symptomatic anterior uveitis should be investigated for the presence of HLA-B27 and examined closely for symptoms of ERA for a differential diagnosis [37].

This study had several limitations. Possible biases may have occurred because of the retrospective, single-center nature of this study. Furthermore, our study was conducted in a subspecialty clinic at a university hospital, and therefore, it does not reflect the entire spectrum of pediatric uveitis that is observed and treated in the community. However, in Korea’s medical system, there are no pediatric ophthalmologists in primary and secondary hospitals; therefore, almost all children diagnosed with uveitis in primary and secondary hospitals are referred to tertiary hospitals. Thus, there may be patients who have not been referred to a tertiary referral center, but considering the healthcare delivery system in Korea, the number is expected to be insignificant. Additionally, the data were compiled from examinations by different ophthalmologists, and some datasets had insufficient information. Many data sources were classified as unknown records, which may have affected the relevance of the results. Furthermore, standardized evaluation and treatment protocols have yet to be established in ophthalmology. Therefore, the choices of treatment and evaluation modalities were greatly influenced by the personal preferences of the ocular inflammation specialists. In addition, pediatric patients in our study are still being followed up and we cannot determine the course because the disease is ongoing. Some patients did not have enough long-term follow-up information. These insufficient data caused inaccuracies in analyzing the course and duration of uveitis. Regardless, we believe that our results add new and important knowledge regarding the epidemiology of pediatric uveitis and the associated systemic diseases.

## Conclusion

In conclusion, systemic rheumatic disease was identified in approximately a third of Korean children with uveitis, and the majority of these patients had JIA. The ANA and HLB-B27 positivity rates were significantly higher in patients with JIA than in those with idiopathic uveitis. Therefore, patients with positive ANA and HLA-B27 results should be referred to a pediatric rheumatologist to confirm the possibility of an association with JIA. A prospective multicenter study would help to further delineate the systemic disease associations and clinical features of pediatric uveitis in Korea.

## Supplementary Information

**Additional file 1.** Classification by JIA subtype.

## Data Availability

All data generated or analyzed during this study are included in this published article and the additional files.

## Abbreviations

JIA: Juvenile idiopathic arthritis
ANA: Antinuclear antibody
HLA: Human leukocyte antigen
DMARDs: Disease-modifying antirheumatic drugs
ILAR: International League of Associations for Rheumatology
ERA: Enthesitis-related arthritis
IQR: Interquartile range
AS: Ankylosing spondylitis
