# Supplementary material for: Epidemiology of pediatric uveitis and associated systemic diseases
Source: Pediatr Rheumatol Online J. 2021 Apr 1;19:48. doi: 10.1186/s12969-021-00516-2 (PMC8015176; doi:10.1186/s12969-021-00516-2)
Supplement: Supplementary file 1 — Additional file 1. Classification by JIA subtype. [file 12969_2021_516_MOESM1_ESM.docx]

**Additional file 1. Classification by JIA subtype**

| JIA subtype | N (%) |
| --- | --- |
| Systemic arthritis | 1 (4.3) |
| Oligoarthritis | 13 (56.5) |
| Polyarthritis | 1 (4.3) |
| Psoriatic | 0 (0.0) |
| Enthesitis-related arthritis | 8 (34.8) |
| Unclassified arthritis | 0 (0.0) |

JIA, juvenile idiopathic arthritis
